# The Statistical Optimisation of Recombinant β-glucosidase Production through a Two-Stage, Multi-Model, Design of Experiments Approach

**DOI:** 10.3390/bioengineering6030061

**Published:** 2019-07-18

**Authors:** Albert Uhoraningoga, Gemma K. Kinsella, Jesus M. Frias, Gary T. Henehan, Barry J. Ryan

**Affiliations:** School of Food Science and Environmental Health, College of Sciences and Health, Technological University Dublin, Dublin D07 ADY7, Ireland

**Keywords:** *Streptomyces griseus*, recombinant β-glucosidase, Fractional Factorial design Plackett-Burman Design, Definitive Screening Design, Response Surface Methodology

## Abstract

β-glucosidases are a class of enzyme that are widely distributed in the living world, with examples noted in plants, fungi, animals and bacteria. They offer both hydrolysis and synthesis capacity for a wide range of biotechnological processes. However, the availability of native, or the production of recombinant β-glucosidases, is currently a bottleneck in the widespread industrial application of this enzyme. In this present work, the production of recombinant β-glucosidase from *Streptomyces griseus* was optimised using a Design of Experiments strategy, comprising a two-stage, multi-model design. Three screening models were comparatively employed: Fractional Factorial, Plackett-Burman and Definitive Screening Design. Four variables (temperature, incubation time, tryptone, and OD_600 nm_) were experimentally identified as having statistically significant effects on the production of *S.griseus* recombinant β-glucosidase in *E. coli* BL21 (DE3). The four most influential variables were subsequently used to optimise recombinant β-glucosidase production, employing Central Composite Design under Response Surface Methodology. Optimal levels were identified as: OD_600 nm_, 0.55; temperature, 26 °C; incubation time, 12 h; and tryptone, 15 g/L. This yielded a 2.62-fold increase in recombinant β-glucosidase production, in comparison to the pre-optimised process. Affinity chromatography resulted in homogeneous, purified β-glucosidase that was characterised in terms of pH stability, metal ion compatibility and kinetic rates for *p*-nitrophenyl-β-D-glucopyranoside (*p*NPG) and cellobiose catalysis.

## 1. Introduction 

Recombinant protein expression has traditionally been an empirical process that required running a large number of experiments to explore many influencing variables (e.g., expression vectors, hosts, expression conditions and media compositions) [1]. The expression of recombinant β-glucosidase, similar to other recombinant proteins, is influenced not only by the expression host strain, but also by expression conditions and media composition [2,3]. β-glucosidase catalyses the hydrolysis of β-1,4-glycosidic bonds, and its industrial applications are well documented [4,5]. However, low yields of this enzyme have been a bottleneck for industrial applications, such as saccharification for biofuels production, and enzymatic synthesis of alkyl-glycosides and oligosaccharides, where large enzyme concentrations are needed [6,7]. In an attempt to address this issue of poor production yields, this study applied a statistical approach, Design of Experiments (DoE), to enhance the production of a recombinant β-glucosidase. The application of DoE to optimise protein production has been recently reviewed [8,9]. Enhanced production of recombinant β-glucosidase, following DoE, has been detailed for recombinant β-glucosidases from a variety of sources, such as *Pichia pastoris* [3], *A. niger* HN-2 [10] and *A. niger* [11]. However, to date, no attempt has been made to enhance the production of β-glucosidase form *Streptomyces* sp.; a species known to be an effective source of β-glucosidase genes [12,13], with limited sequence conservation compared to *P. pastoris* (18% conservation) and *A. niger* (26% conservation). The aim of this study was to optimise the production of *S. griseus* recombinant β-glucosidase in *E.coli* BL21 (DE3) by using multiple screening designs to validate the variable selected for optimisation via a response surface methodology. The findings of this study are explored in light of the existing literature and recommendations are offered for future applications of Design of Experiments to enhance recombinant protein production.

## 2. Materials and Methods

### 2.1. Chemical and Materials

The recombinant pGEX-4T-1 vector containing *S. griseus* β-glucosidase gene (GST-tagged) in *E. coli* BL21 (DE3) was developed in a previous study [14]. Ampicillin, glycerol, Isopropyl-β-D-thiogalactopyranoside (IPTG), LB broth, *p*-nitrophenyl-β-D-glucopyranoside (*p*NPG), *p*-nitrophenol (*p*NP), cellobiose, fructose sucrose, tryptone, yeast extract, beef extract, CaCl_2_, DTT, KOH, MgCl_2_, (NH_4_)_2_S_4_, ZnSO_4_, Triton X-100, M PMSF, Lysozyme, Bradford reagent, and Glutathione Sepharose 4B resin were purchased from Sigma Aldrich (Ireland). 

### 2.2. Production of S. griseus Recombinant β-glucosidase 

#### 2.2.1. Preparation of Microbiological Media

The recombinant pGEX-4T-1 vector containing *S. griseus* β-glucosidase gene (GST-tagged) in *E. coli* BL21 (DE3) was inoculated into 5 mL of Luria-Bertani (LB) medium containing ampicillin at a final concentration of 50 µg·mL^−1^, and incubated at 37 °C for overnight at 220 rpm. The overnight culture was used to inoculate 10% v/v of fresh medium for small-scale (10 mL) expression studies used during the screening and optimisation processes. 

#### 2.2.2. Carbon and Nitrogen Sources for *S. griseus* Recombinant β-glucosidase Expression

Identification of good carbon and nitrogen sources for *S. griseus* recombinant β-glucosidase expression was initially performed. Four carbon sources (glucose, fructose, galactose, and glycerol) and six nitrogen sources (yeast extract, tryptone, beef extract, KNO_3_, NH_4_Cl, and (NH_4_)_2_SO_4_) were selected based on their noted effect on recombinant expression in *E-coli* in general [15,16], and on β-glucosidase in particular [17,18]. The effect of carbon and nitrogen sources was investigated in basal medium supplemented with 0.5% (w/v) of the different carbon sources and 1% (w/v) of the different nitrogen sources [19]. In each case, cells were grown until OD_600 nm_ reached 0.5, and then induced with 1mM IPTG for 6 h with subsequent culturing at 37 °C, 220 rpm. 

#### 2.2.3. Effect of Culture Aeration on *S. griseus* Recombinant β-glucosidase Expression 

Subsequently, the influence of culture aeration, via baffled culture flasks, on the protein expression was also investigated by cultivating cells with medium supplemented with the selected carbon and nitrogen sources, 0.5% w/v fructose, 1% w/v yeast extract and tryptone (see Section 2.2.2), in baffled and non-baffled flasks, at 37 °C, 220 rpm for 6 h post 1 mM IPTG induction.

#### 2.2.4. Screening of Most Significant Medium Components and Induction Condition Using Multiple Screening Designs 

Fractional Factorial Design (FFD), Plackett-Burman Design (BBD), and Definitive Screening Design (DSD) were employed to comparatively screen for the most significant medium components and induction conditions affecting *S. griseus* recombinant β-glucosidase production in *E. coli* BL21 (DE3). A total of seven factors, including four induction related conditions (OD, IPTG, temperature, incubation time) and three medium components (yeast extract, tryptone, fructose) were considered for the screening experiments. All factors were studied at two levels; high and low, denoted by (+) and (−) signs, respectively (see Table 1). The FFD was created using the main effects only and confounding all interactive effects by using Resolution 4 within *JMP*. For the DSD, two centre-point replicates were used to verify the variation in the screening process and to validate that the two level design linear assumption was true. The multiple screening designs used in this study, along with their responses, are summarised in Table 2, Table 3 and Table 4. A statistical analysis of the experimental data was performed and a densitometry analysis of sodium dodecyl sulfate polyacrylamide gel electrophoresis (SDS-PAGE) of the expressed β-glucosidase was carried out using *ImageJ* densitometry software, http://rsb.info.nih.gov/ij/ [20].

#### 2.2.5. Optimisation of *S. griseus* Recombinant β-glucosidase Production by Response Surface Methodology

A Central Composite Design (CCD) was applied to identify the optimum levels of the most effective variables (temperature, induction time, tryptone, and OD_600 nm_) previously identified in the screening process. Each variable in the design was examined at three levels, low (−), central (0), and high (+; see Table 5). 

Central Composite Design was the preferred Response Surface Methodology, due to the fact that this design permitted full, or fractional, factorial modes, with the potential to add central points to evaluate the experimental error [21]. In this experiment, the total number of runs was calculated using Equation (1).

(1) $$N=k^{2}+2k+Cp$$

where *k* is the number of factors and *Cp* the number of centre points [22]. An experimental design by CCD was developed with a total number of 28 runs, including four replicates at the central point. The full experimental plan comprising maximum, central and minimum ranges of the screened variables is provided in Table 6. 

#### 2.2.6. Statistical Analysis 

*JMP 13* (SAS Institute, Wittington House, UK) was utilised to design experiments and to analyse, through regression analysis, the experimental data. The response obtained (BGL activity: U/mL) was also subjected to ANOVA. A second-order polynomial equation was then fitted to the data using a multiple regression procedure (Equation (2)).

(2) $$Y=\;\beta 0\;+\;\sum \beta_{i}X_{i}+\;\sum \beta_{ii}X_{i}^{2}+\sum \beta_{ij}X_{i}X_{j}$$

where Y is the predicted response, β0, βi, βii, and βij are coefficients for the intercept, linear, square or quadratic, and interactive terms, respectively. *Xi* and *Xj* are the independent variables [23]. The fit of the model was also evaluated through ANOVA (see Table 7). The coefficient value (R^2^) was used to define how well the data fit the model used; whilst the *p*-value and “lack of fit” were used to estimate the appropriateness of the model [24]. The significance of regression coefficients were also examined (see Table 8). Finally, the experimental and predicted values were compared to determine the validity of the developed model. 

#### 2.2.7. Optimum Determination and Validation 

To determine the optimum factor levels for maximal yields of recombinant β-glucosidase, surface contour plots were utilised. In these 3-D plots, two test factors were utilized, whilst the other factors were maintained at their respective zero levels [25]. The optimum conditions were verified by conducting validation experiments comprising three independent experiments examining the responses generated in comparison to the model-predicted results.

#### 2.2.8. Purification of *S. griseus* Recombinant β-glucosidase by Affinity Chromatography

Following six hours of induced *S. griseus* recombinant β-glucosidase, the cell mass was pelleted by centrifugation at 7000 ×*g* for 10 min at 4 °C. The pellet was lysed by resuspension in lysis buffer (10 mM Na_2_HPO_4_, 1.8 mM KH_2_PO_4_, 140 mM NaCl, 1% (v/v)) Triton X-100, 1mM DTT, 1mM PMSF, 10 mg·mL^−1^ Lysozyme) and sonication at an amplitude of 40 for 30 s using an Ultrasonic processor Sonicator (QSONICA, Newtown, CT, USA). The resultant slurry was centrifuged at 14,000 ×*g* for 30 min at 4 °C and the cleared lysate was further filtered through an Amicon^®^ Ultra centrifugal filter to concentrate the lysed protein mixture. The concentrated lysed protein mixture was loaded onto a Glutathione Sepharose 4B resin column and incubated for 30 min at 4 °C with gentle shaking. Subsequently, the *S. griseus* recombinant β-glucosidase was fractionally collected at a flow rate of 0.5 mL/min, using a gravity flow column. The GST-tag was on-column cleaved from the purified *S. griseus* recombinant β-glucosidase by *PreScission* protease using a washing (50 mM Tris, 150 mM NaCl, pH 8) and elution buffer (50 mM Tris, 150 mM NaCl, 12 mM reduced glutathione, pH 8) combination [26]. The eluted enzyme was dialysed against 1.0 L of 50 mM potassium phosphate buffer, pH 7 at 4 °C for 24 h with constant, gentle stirring. The protein purity was verified by 10% (v/v) SDS-PAGE [27].

#### 2.2.9. Determination of Protein Concentration 

The protein concentration was determined according to the Bradford Method [28], using an adapted 96-well plate approach. Bovine serum albumin was used as standard, with a working protein concentration linear range of 0 to 1 mg·mL^−1^, in 50 mM Potassium phosphate buffer (pH 7). The protocol entailed, in triplicate, 5 µL of the protein standard/diluted sample was added to an individual well. The negative control was 5 µL of the buffer (50 mM Potassium phosphate, pH 7) in place of the protein standard/diluted sample. Subsequently, 250 µL of the Bradford reagent (Sigma) was added to each well sequentially, using a multichannel pipette, and the mixtures were thoroughly mixed by using the mixing cycle on the microplate spectrophotometer (Bio-Tek PowerWave) for 10 s and incubated at room temperature for 20 min. The resulting absorbance was measured at 595 nm against the blank and the absorbance of the unknown protein concentration was determined in comparison to the standards. 

#### 2.2.10. Determination of *S. griseus* Recombinant β-glucosidase Activity 

The recombinant β-glucosidase activity was measured using a standard enzyme activity assay by determining the hydrolysis of the substrate *p*-nitrophenyl-β-D-glucopyranoside (*p*NPG) using a 96-well-plate-based protocol. In brief, 20 µL the purified recombinant β-glucosidase (0.5 mg·mL^−1^) was mixed with 120 µL potassium phosphate buffer (50 mM, pH 7) and 30µL *p*NPG (7 mM) substrate, and incubated at 37 °C for 20 min. A negative control comprised the same components with the enzyme volume replaced with buffer. The reaction was terminated by adding 30 µL of 1 M Na_2_CO_3_ and the total reaction volume was 200 µL. The release of *p*-nitrophenol (*p*NP) was measured at 405 nm using a microplate spectrophotometer (Bio-Tek PowerWave). All experiments were performed as three independent experimental runs, themselves as triplicates. The colour developed was translated to µmol *p*NP using a standard curve in the range of 0 to 0.5 mM, prepared as outlined [29]. One unit (IU) of β-glucosidase activity was defined as the amount of enzyme required to release 1 µmol of product (*p*NP) per minute under the standard assay conditions.

#### 2.2.11. Stability Studies and Characterisation 

Characterisation studies, unless otherwise stated, utilised the standard enzyme assay (see Section 2.2.10). To examine the effect of pH on the β-glucosidase activity, the pH stability was performed by incubating the enzyme in the reaction buffers: citrate buffer (pH 5.0), phosphate buffer (pH 6.0–8.0) at 37 °C for 0 to 180 min [30]. The effect of metal ions on the enzyme was determined using 1 mM of several metal ions (Ca^2+^, Mg^2+^_,_ N^+^, K^+^, ZnSO_4_, and (NH_4_)_2_SO_4_) at 37 °C, for 60 min and 240 min. The kinetic parameters were determined from Michaelis–Menten plots of reaction rate of three independent experiments at 37 °C, using varying *p*NPG substrate concentrations between 4 mM to 32 mM, and cellobiose from 5 mM to 50 mM, under standard assay conditions. For cellobiose, the determination of glucose released was measured using a Colourimetric Assay Kit (Invitrogen, Thermo Scientific, Cork, Ireland), according to manufacturer’s instructions. The values of V_max_ and K_m_ were determined by non-linear regression analysis, using GraphPad Prism (Version 7, GraphPad Software, San Diego, CA, USA).

## 3. Results and Discussion

Recombinant β-glucosidases from different sources, heterologously expressed in prokaryotic and eukaryotic systems have typically utilised the traditional One Factor At a Time (OFAT) method to optimise yield [18]. However, this classical approach requires a significant number of experiments and fails to account for interactions between variables, which can result in low yields [8,31]. In this study, the production of *S. griseus* recombinant β-glucosidase has been optimised, instead, by Design of Experiments with the emphasis on optimising parameters that affected expression. Prior to Design of Experiments, preliminary investigations on carbon and nitrogen sources, along with the effect of aeration, were performed to reduce the number of factors to be explored through DoE. This permitted the comparative screening process to cover a more targeted experimental space, facilitating the selection of the most influential factors, whilst simultaneously ensuring a validated screen and effective subsequent optimisation [32]. 

### 3.1. Effect of Carbon and Nitrogen Sources 

**Figure 1:**
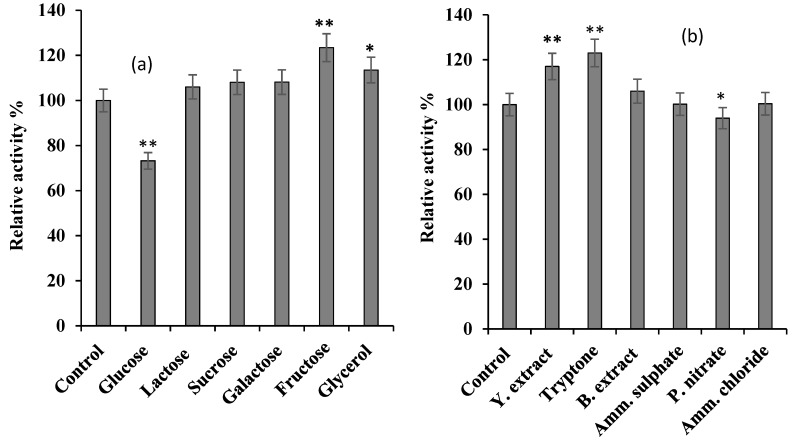
The effect of various carbon (**a**) and nitrogen (**b**) sources on *S. griseus* recombinant β-glucosidase expression. Recombinant β-glucosidase was expressed by supplementing basal medium with 0.5% (w/v) and 1% (w/v) of different carbon and nitrogen sources, respectively. The control was performed using only basal medium. In each case, cells were grown until OD_600 nm_ reached 0.5, and then induced with 1 mM IPTG for 6 h at 37 °C, 220 rpm. Enzyme activity of crude lysate was performed using 7 mM *p*NPG as the substrate in 50 mM potassium phosphate buffer, pH 7 (see Section 2.2.2). The data plotted represent the mean of three independent experiments, with standard deviation shown as error bars: * *p*-value ≤ 0.05, ** *p*-value ≤ 0.01 represent a significant and a very significant difference, respectively, based on two-tailed *t-*test and in comparison to the control.

Carbon is important to all living organisms and the breakdown of the carbon source liberates energy, which is utilised by the organism for growth and development [33]. The most commonly used carbon sources in heterologous protein expression are glucose, starch, glycerol, fructose, maltose, arabinose, sucrose, lactose, and xylose [33,34,35,36]. In this study, the effect of six different carbon sources (glucose, lactose sucrose, galactose, fructose and glycerol) on the expression of *S. griseus* recombinant β-glucosidase was investigated (see Figure 1a). As expected, glucose was found to significantly repress expression, by 23% (*p*-value ≤ 0.01); the repressive effect of glucose on β-glucosidase could be the catabolic repression of glucosidase synthesis, which was reported in literature [37,38]. In contrast, fructose significantly enhanced the expression of *S. griseus* recombinant β-glucosidase, by 17% (*p*-value ≤ 0.01), and echoes previous studies [39]. 

Nitrogen sources have been previously reported to influence the production of protein in general [40] and β-glucosidase in particular [17]. In this study, the effect of six nitrogen sources (yeast extract, tryptone, beef extract, ammonium sulfate, ammonium chloride, and potassium nitrate) on *S. griseus* recombinant β-glucosidase were explored (see Figure 1b). Tryptone and yeast extract exhibited a significant effect (*p*-value ≤ 0.01) on the expression of *S. griseus* recombinant β-glucosidase; 23% and 17% increase in production, respectively, mirroring previous reports [18,41]. In contrast, potassium nitrate exhibited a significant repressive effect by 15% (*p*-value ≤ 0.05) of production, while beef extract, ammonium sulphate, and ammonium chloride did not show any significant effect on the production of this recombinant enzyme.

### 3.2. Effect of Aeration on S. griseus Recombinant β-glucosidase Expression 

**Figure 2:**
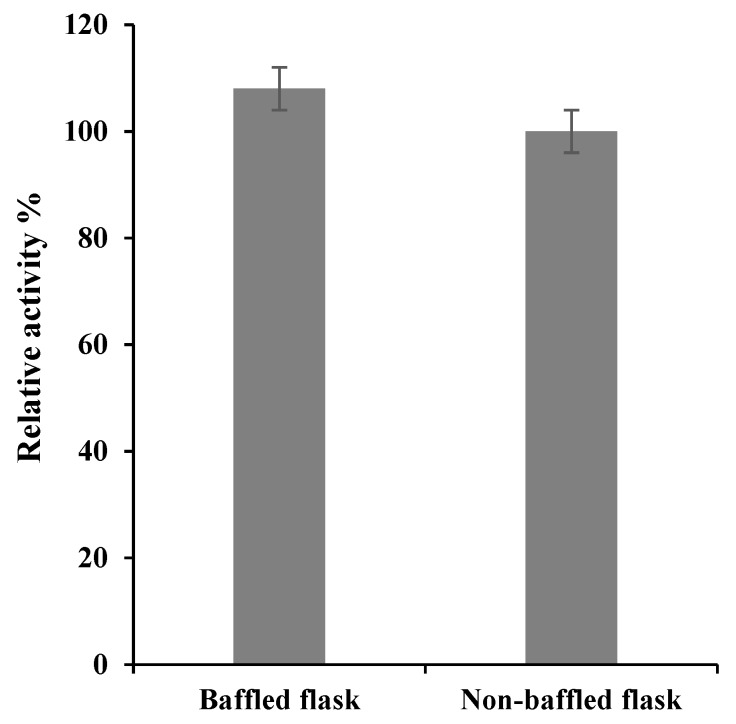
The effect of baffled versus non-baffled flask on the production of *S. griseus* recombinant β-glucosidase. Recombinant β-glucosidase was expressed in basal medium supplemented with 0.5% (w/v) fructose, 1% (w/v) yeast extract and tryptone at 37 °C for 6 h, following 1 mM IPTG induction. Enzyme activity was used as a proxy to enzyme production. The data represented are the mean of three independent experiments, with the standard deviations noted as error bars. Both baffled and non-baffled flasks show no significant difference between them (*p*-value ≥ 0.235) based on two-tailed *t-*test.

Aeration is an important parameter known to effect recombinant protein expression and baffled flasks are a common approach to enhance protein synthesis through enhanced oxygenation efficiency [42]. To understand the effect of aeration on expression of *S. griseus* recombinant β-glucosidase, the expression was carried in baffled and non-baffled flasks with basal medial supplemented with optimum carbon and nitrogen sources (Section 3.1). No statistically significant difference was noted between a baffled and non-baffled flaks culture (see Figure 2) and chimes with results previously reported on the production on β-glucosidase in *Pichia pastoris* [3]. Baffled culture phenomena, such as foaming [43] or reduced metabolite production [44,45], may result in similar yields to a non-baffled culture.

### 3.3. Screening of most Significant Media Components and Induction Conditions

**Figure 3:**
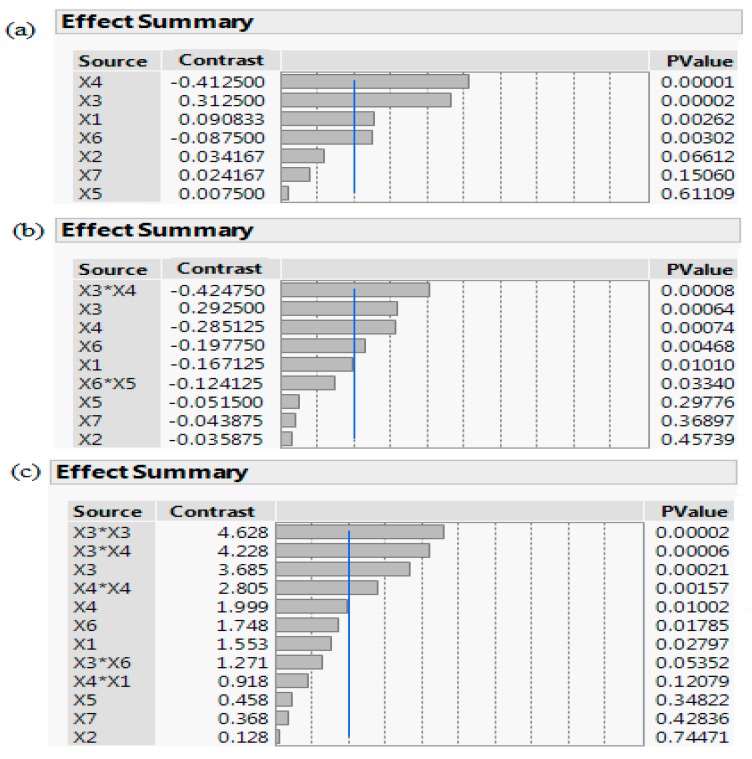
A pareto chart of the screening processes: (**a**) PBD, (**b**) FFD, and (**c**) DSD. The schematic depicts a scaled summary and corresponding *p*-value of the seven factors: OD (Abs_600 nm_) at induction time (X_1_), IPTG (X_2_), temperature (X_3_), incubation time (X_4_), yeast extract (X_5_), tryptone (X_6_), fructose (X_7_), along with their interactions. Factors with *p*-value < 0.05 are statistically significant and variables crossing the reference blue line with *p*-value < 0.001 are considered as highly statistically significant.

**Figure 4:**
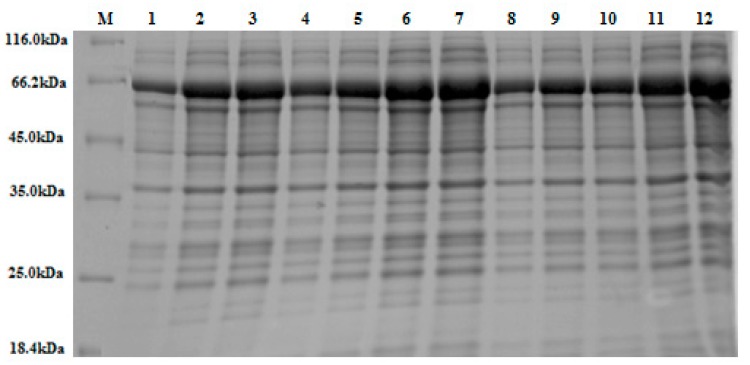
A 10% (w/v) SDS-PAGE stained with Coomassie Brilliant Blue. The figure depicts various band sizes of crude β-glucosidase expressed in accordance with the 12 experimental runs, according to Plackett-Burman Design (see Section 2.2.4). An equal volume of crude extract (15 μL) was loaded into each lane. Lanes 2, 3, 6, 10, 11, and 12 display larger over-expressed protein bands at the expected molecular weight for the recombinant β-glucosidase/GST fusion protein (~65 kDa), as based on ImageJ densitometric analysis. This result mirrors the increased enzyme activity (U/mL) observed in these experimental runs (see Table 2). Note: M = protein marker (14.4–116 kDa).

Multiple screening designs (PBD, FFD, DSD) were simultaneously used to identify the most influential variables and validate the reliability of the screening matrix through inter-screen correlation. The average response ranged from 39.21 U/mL to 41.24 U/mL (see Table 2, Table 3 and Table 4), providing a rationale to optimise the medium constituents and induction conditions for maximal *S. griseus* β-glucosidase production. Experimental data were statistically interrogated to identify and categorise the most influential variables (see Figure 3). In all cases, the multiple screening processes identified the same important factors as most influential; temperature (X_3_) and incubation time (X_4_) were highly statistically significant (*p*-value < 0.001), whereas tryptone (X_6_) and OD_600nm_ (X_1_) were statistically significant (*p*-value < 0.05). These results align with previous studies that employed factorial design to enhance β-glucosidases expression [46,47]. Other factors, namely IPTG (X_2_), yeast extract (X_5_) and fructose (X_7_), were not statistically significant (*p*-value > 0.05). The interaction effect of X_3_*X_4_, and the quadratic effects of X3*X3, X4*X4, were found to be highly significant (*p*-value < 0.001; see Figure 3b,c), indicating that these factors interact, and any change in one would affect the other, as well as the response. These affects were also visualized via SDSPAGE (see Figure 4).

### 3.4. Optimisation of Screened Variables for Maximal Production

A three level Central Composite Design was used to optimise the production of *S. griseus* recombinant β-glucosidase. Table 4 details the experimental design and corresponding response (actual, predicted, and residuals) of the production β-glucosidase.

**Figure 5:**
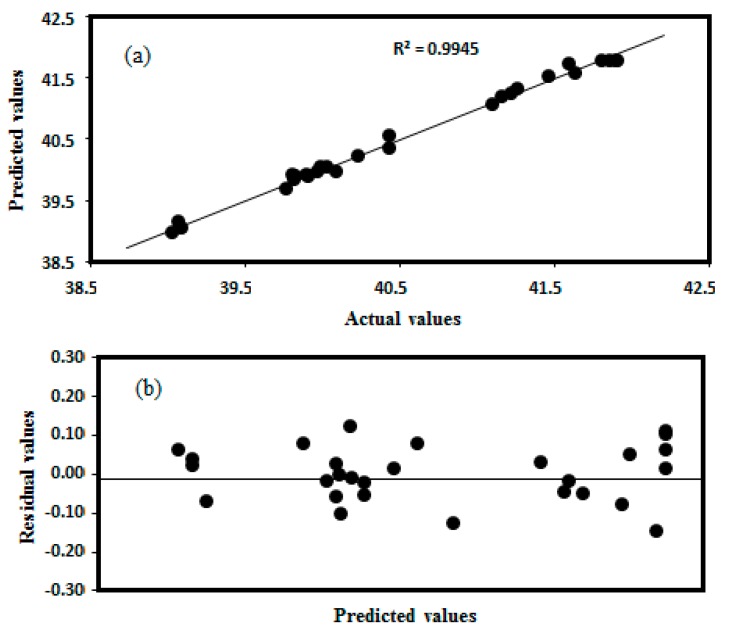
A plot of (**a**) actual versus predicted, and (**b**) predicted versus residual values to estimate the accuracy of the regression model.

The model adequacy was verified by multiple regression analyses, utilising a second-order polynomial fitted to Equation (3).

(3) $$\begin{array}{ll} Y=41.7891- & 0.0566\;X1-0.2557\;X3-0.1524\;X4\;+0.0754\;X6\;-0.5328\;X1*X1\\ & -0.9758\;X3*X3-0.4068\;X4*X4\;-0.1308\;X6*X6\;+0.0133\;X1*X3\\ & +0.0014\;X1*X4\;+0.0008\;X1*X6-0.285\;X3*X4-0.0557\;X3*X6\\ & +0.0239\;X4*X6 \end{array}$$

where Y is response, X_1_ is OD_600 nm_, X_3_ is Temperature, X_4_ is Incubation time, X_6_ is Tryptone. The data were analysed by analysis of variance (ANOVA, see Table 7), with the model F-value noted as being highly significant (*p*-value < 0.001). The model “goodness of fit” (*R^2^*_adjusted_ = 0.9885) confirmed the appropriateness of the model to predict the response [48]. The “model lack of fit” F-value was not significant (6.87; *p*-value > 0.05), confirming the accuracy of the model [49]. The predicted *R^2^* (0.9945) indicates a good agreement between the value predicted by the model and the experimental data (see Figure 5a). A plot of residual values versus predicted values also revealed no trends (see Figure 5b), implying homogeneity of variance in the data and absence of outliers in the experimental runs [25].

The regression coefficient significance under a student t-test (see Table 8) indicated that temperature and incubation time were constantly found to be the most influential factors and highly significant (*p*-value < 0.001). All of the square terms, except X_6_*X_6_, were also found to be highly significant (*p*-value < 0.001). Interaction coefficients were significant with the order of X_3_*X_4_ > X_3_*X_6_, indicating the importance of the interacting variables.

**Figure 6:**
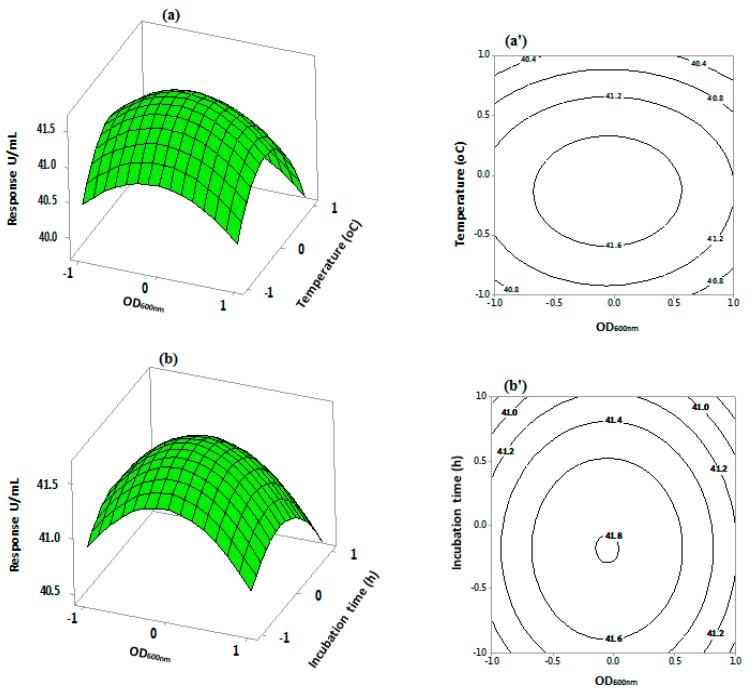

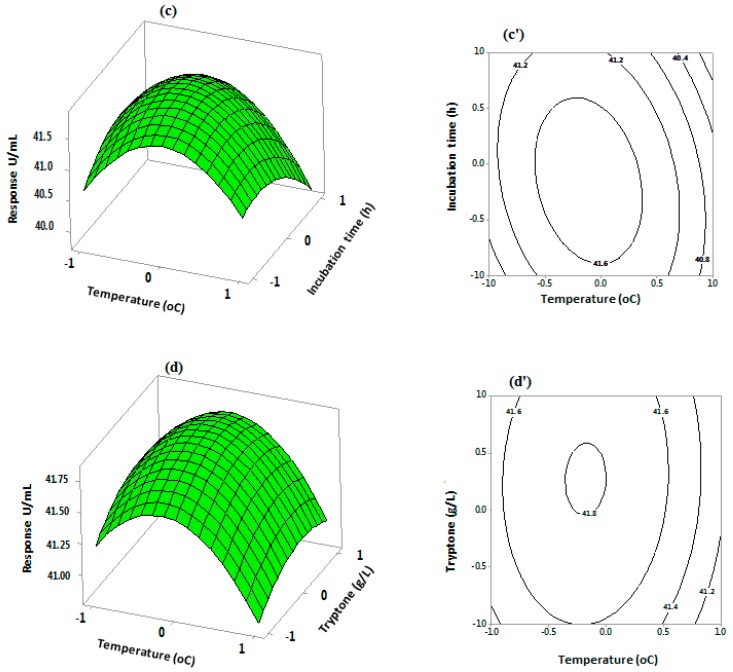
Three-dimensional response surface and contour plots for *S.griseus* recombinant β-glucosidase production using a three level Central Composite Design. The plots depict the interactive effects of: OD_600 nm_ and temperature (**a**,**a’**); OD_600 nm_ and incubation time (**b**,**b’**); temperature and incubation time (**c**,**c’**); and temperature and tryptone (**d**,**d’**). The remaining variables remained at constant zero levels.

Three dimensional response surface and contour plots [50,51] were used to predict optimum factor levels for maximal production of *S.griseus* recombinant β-glucosidase (see Figure 6). The response surface and contour plots between OD_600 nm_ and temperature (Figure 6a,a’), and OD_600 nm_ and incubation time (Figure 6b,b’), displayed curved relationships, indicating these variables significantly influenced the production of *S. griseus* recombinant β-glucosidase, with maximum enzyme activity observed at central variable levels. The response surface and contour plots between temperature and incubation time (Figure 6c,c’) indicate a direct correlation between the production of *S. griseus* recombinant β-glucosidase and both variables. The maximum enzyme activity was determined at central levels and decreased at the extreme levels. The relationship between temperature and tryptone presented an elliptical shape and was significant (see Figure 6d,d’ and Table 6). Increasing the tryptone concentration (to 15 g/L) resulted in increased enzyme production, with the optimum noted at temperature central point. A relationship between the 3D response surface and the statistically significant factors (at *p*-value < 0.05) optimised in this study indicates that the statistical model developed was appropriate to cover all independent variable ranges investigated in this study.

### 3.5. Validation of Central Composite Design for Optimisation

Experiments were carried out independently at the identified optimal levels of the statistically significant variables (OD_600 nm_, 0.55; temperature, 26 °C; incubation time, 12 h; and tryptone, 15 g/L) and at the middle levels of the other variables to verify the validity of the optimisation model. Subsequently, the experimental results were compared with the predicted results and a control experiment utilising the pre-optimised process parameters in basal medium. The observed value of enzyme activity (41.900 U/mL) was in good agreement with the predicted value (41.789 U/mL; see Table 6). The CCD optimisation of *S. griseus* recombinant β-glucosidase production reached 42 U/mL enzyme activity, representing a 2.62-fold increase in β-glucosidase production when compared to pre-optimised conditions (see Table 9). This fold increase in production is similar to production increases for recombinant β-glucosidases from different sources following DoE –based optimization, 2.21-fold [30] and 2.2-fold [10]; however, larger fold increases, up to 5.7-fold, have also been reported [46].

### 3.6. Affinity Purification of S. griseus Recombinant β-glucosidase

**Figure 7:**
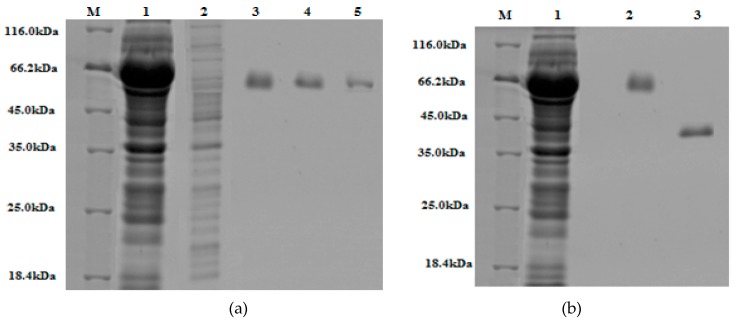
The 10% (w/v) SDS-PAGE gels stained with Coomassie Brilliant Blue: (**a**) Lane M, protein marker (18.4–116 kDa); lane 1, crude extract; lane 2, run-through; lane 3, 4, 5, are elute one, two, and three of purified GST-tagged β-glucosidase, respectively. (**b**) Lane M, protein marker (18.4–116 kDa); lane 1, crude extract; lane 2, purified GST-tagged β-glucosidase; lane 3, purified β-glucosidase (post GST-tag cleavage).

An overall yield of 47%, with a specific activity of 9.13 U/mg against *p*NPG as the substrate, was noted following Glutathione S-Transferase (GST) tag purification and on-column tag cleavage. Successful purification was verified through SDS-PAGE analysis (Figure 7a) and activity assay (see Table 10). The final purified enzyme showed a single band with a molecular mass of approximately 42kDa (Figure 7b).

### 3.7. Fundamental Characterisation

**Figure 8:**
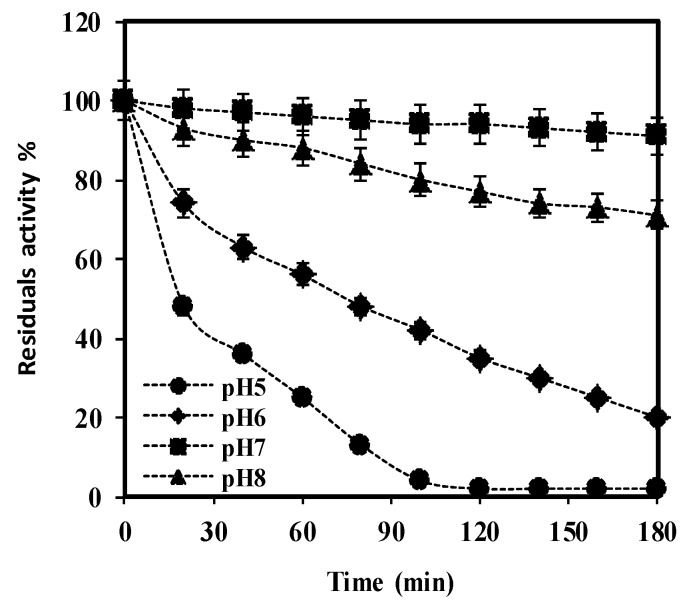
The pH stability of purified *S. griseus* recombinant β-glucosidase was determined by incubating enzyme solutions containing 7 mM pNPG in buffers of various pH, ranging between 5 (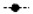), 6 (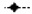), 7 (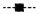), and 8 (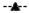), over a period of 180 min at 37 °C. Residual activity (%) at each time point was calculated considering the initial activity, at zero time as 100%. Data represent the mean of three independent experiments, with standard deviations noted as error bars.

The purified *S. griseus* recombinant β-glucosidase and commercial almond β-glucosidase (Sigma) were previously used to evaluate the effect of pH, effector molecules, and natural substrate, and have been documented [14]. Here, following detailed pH stability profiling, purified *S. griseus* recombinant β-glucosidase was noted to retain >95% activity at pH 7, decreasing to 78% at pH 8 over a period of 180 min, and 50% at pH 6 after 75 min at 37 °C (see Figure 8). An optimal range of pH 6 to 8 is a common feature of β-glucosidase enzymes isolated from diverse bacterial strains [52,53,54,55,56]. 

### 3.8. Effect of Metal Ions and Chemical Reagent on Purified S. griseus β-glucosidase 

Metal ions can elicit inhibitory effects β-glucosidase [57,58]. Several metal ions were examined (see Table 11), with modest activation noted in Ca^2+^, Mg^2+^, N^+^, and K^+^ after 6 h incubation. These results echo reports of β-glucosidase enhancement, through conformational change, by Ca^2+^ and Mg^2+^ ions [59,60].

### 3.9. Kinetic Parameters

Two substrates (synthetic *p*NPG, and natural cellobiose) were used to determine the kinetic parameters for the purified recombinant *S.griseus* β-glucosidase (see Table 12). A higher affinity for a synthetic substrate in comparison to a natural β-glucosidase substrate has been previously reported for β-glucosidases from *Thermoanaerobacterium* [56] and *Phoma sp* KCTC11825BP [61]. The high K_m_ and V_max_ indicated that this enzyme has less affinity for *p*NPG and cellobiose compared to other reports for cellobiose, with K_m_ 1.0 mM, V_max_ 144 μmol·min^−1^·mg^−1^ [62], and for *p*NPG, with K_m_ 3.3 mM, V_max_ 43.68 μmol·min^−1^·mg^−1^ [63]. Further kinetic parameters on β-glucosidases from various sources are given in Table 13.

## 4. Conclusions

The production of functional recombinant enzymes in sufficient concentration for industrial applications is often a bottle-neck. Production optimisation can, however, result in significant increases in yield. Here, the statistical Design of Experiments approach was used as an efficient technique to identify the key factors and levels required for optimised production of *S.griseus* recombinant β-glucosidase in *E. coli* BL21 (DE3). The use of multiple screening designs in this study internally validated the selection of the most influential factors as temperature and incubation time, followed by tryptone and OD_600 nm_ for induction. These variables were optimised, through a Central Composite Design, resulting in a 2.62-fold increased yield. Previous characterisation data were supplemented in this study and concluded that the *S. griseus* recombinant β-glucosidase purified enzyme exhibited optimum activity at pH 7, had a temperature optimum of 69 °C and displayed increased activity in the presence of Mg^2+^, N^+^, Ca^2+^_,_ and K^+^, whilst it had a higher affinity for the artificial substrate *p*NPG in comparison to the natural cellobiose substrate.

With this increased production capacity, a more detailed understanding of stability and substrate specificity, this *S. griseus* recombinant β-glucosidase could be useful for a variety of applications, including hydrolysis of biomass into fermentable sugars, hydrolysis of lactose during processing lactose containing products, and enzymatic synthesis of alkyl glycosides, where β-glucosidases of similar characteristics have been reported to be useful [7,18,71,72].

## Figures and Tables

**Table 1:** The minimum and maximum ranges for different factors studied during screening process.

Factors Types	Factors Codes	Factors	Levels	
			−1 (Low)	+1 (High)
Induction condition	**X**_1_	OD (Abs_600 nm_)	0.3	0.9
	X_2_	IPTG (mM)	0.1	1.0
	X_3_	Temperature (°C)	18	36
	X_4_	Incubation time (h)	4.0	20
Media Composition	X_5_	Yeast Extract (g/L)	5.0	10
	X_6_	Tryptone (g/L)	10	20
	X_7_	Fructose (g/L)	1.0	5.0

**Table 2:** Plackett-Burman Design. Multiple screening designs for identification of most influential factors on the production of *S.griseus* recombinant β-glucosidase. It depicts coded values of the designs, along with the observed experimental response (β-glucosidase activity (BGL); U/mL).

	Coded Values							Response
Runs	X_1_	X_2_	X_3_	X_4_	X_5_	X_6_	X_7_	BGL (U/mL)
1	1	1	1	1	1	1	1	39.301
2	−1	−1	1	−1	−1	1	−1	39.781
3	−1	1	−1	−1	1	−1	1	40.681
4	1	−1	−1	1	−1	1	1	39.761
5	−1	−1	1	−1	1	1	1	39.801
6	1	1	−1	−1	−1	1	−1	40.631
7	1	−1	1	1	1	−1	−1	39.271
8	−1	1	−1	1	1	1	−1	39.621
9	−1	1	1	1	−1	−1	−1	39.181
10	1	1	1	−1	−1	−1	1	40.211
11	−1	−1	−1	1	−1	−1	1	39.811
12	1	−1	−1	−1	1	−1	−1	40.791

**Table 3:** Fractional Factorial Design. Multiple screening designs for identification of the most influential factors on the production of *S.griseus* recombinant β-glucosidase. It depicts coded values of the designs, along with the observed experimental response (β-glucosidase activity (BGL); U/mL).

	Coded Values							Response
Runs	X_1_	X_2_	X_3_	X_4_	X_5_	X_6_	X_7_	BGL (U/mL)
1	1	1	1	−1	−1	−1	−1	41.641
2	−1	−1	−1	−1	−1	−1	−1	40.471
3	1	−1	−1	1	1	−1	−1	40.571
4	1	−1	−1	−1	−1	1	1	39.821
5	1	−1	1	−1	1	−1	1	41.601
6	−1	1	1	1	1	−1	−1	40.561
7	1	1	1	1	1	1	1	39.411
8	−1	−1	−1	1	1	1	1	40.388
9	1	−1	1	1	−1	1	−1	40.281
10	1	1	−1	−1	1	1	−1	39.631
11	−1	1	−1	1	−1	1	−1	40.421
12	1	1	−1	1	−1	−1	1	40.311
13	−1	−1	1	−1	1	1	−1	41.379
14	−1	1	−1	−1	1	−1	1	40.651
15	−1	1	1	−1	−1	1	1	41.691
16	−1	−1	1	1	−1	−1	1	40.380

**Table 4:** Definitive Screening Design. Multiple screening designs for identification of most influential factors on the production of *S.griseus* recombinant β-glucosidase. It depicts coded values of the designs, along with the observed experimental response (β-glucosidase activity (BGL); U/mL).

	Coded Values							Response
Runs	X_1_	X_2_	X_3_	X_4_	X_5_	X_6_	X_7_	BGL (U/mL)
1	−1	1	1	0	−1	−1	−1	40.111
2	0	0	0	0	0	0	0	41.221
3	−1	0	1	1	1	1	−1	39.033
4	−1	1	0	1	−1	−1	1	40.621
5	1	−1	1	1	0	−1	−1	39.121
6	−1	−1	1	−1	−1	1	1	39.961
7	1	−1	−1	0	1	1	1	40.511
8	1	1	−1	1	1	−1	−1	40.611
9	1	1	1	−1	1	−1	1	40.261
10	1	1	−1	1	−1	1	0	40.011
11	1	0	−1	−1	−1	−1	1	40.561
12	−1	1	−1	−1	0	1	1	39.451
13	−1	−1	−1	1	1	−1	1	40.651
14	0	1	1	1	1	1	1	39.106
15	−1	−1	−1	1	−1	1	−1	39.981
16	−1	1	−1	−1	1	0	−1	39.791
17	1	1	1	−1	−1	1	−1	40.021
18	1	−1	1	1	−1	0	1	39.190
19	−1	−1	1	−1	1	−1	0	39.841
20	1	−1	0	−1	1	1	−1	41.211
21	0	−1	−1	−1	−1	−1	−1	39.781
22	0	0	0	0	0	0	0	41.231

**Table 5:** The most influential factors (X_1_, X_3_, X_4_, X_6_), as identified through the screening process, were examined at three levels; low (−1), central (0), and high (+1), for the optimisation of *S. griseus* recombinant β-glucosidase expression in *E. coli* BL21 (DE3).

Factor code	Factors (unit)	Levels		
		−1	0	+1
X_1_	OD (Abs_600 nm_)	0.3	6.0	0.9
X_3_	Temperature (°C)	18	27	36
X_4_	Incubation time (h)	4	12	20
X_6_	Tryptone (g/L)	10	15	20

**Table 6:** CCD for production optimisation of *S. griseus* recombinant β-glucosidase. The table depicts coded values, along with the experimental response: BGL (U/mL), actual, predicted, and residuals (the difference between the actual and predicted values).

	Coded values				Response: BGL (U/mL)		
Runs	X_1_	X_3_	X_4_	X_6_	Actual	Predicted	Residuals
1	0	0	0	−1	41.633	41.583	0.050
2	−1	1	1	−1	39.072	39.049	0.023
3	1	−1	−1	1	39.899	39.903	−0.004
4	−1	0	0	0	41.263	41.313	−0.050
5	1	1	1	1	39.089	39.052	0.037
6	0	0	1	0	41.213	41.230	−0.017
7	−1	−1	1	1	40.433	40.354	0.079
8	−1	−1	−1	1	39.988	40.043	−0.055
9	1	−1	1	−1	39.804	39.907	−0.103
10	1	−1	1	1	40.232	40.218	0.014
11	1	1	−1	−1	39.824	39.883	−0.059
12	0	1	0	0	40.430	40.558	−0.128
13	0	0	0	0	41.804	41.789	0.015
14	1	1	1	−1	39.023	38.963	0.060
15	0	0	0	1	41.587	41.734	−0.147
16	0	0	0	0	41.900	41.789	0.111
17	−1	1	−1	−1	39.965	39.974	−0.009
18	0	−1	0	0	41.100	41.069	0.031
19	1	0	0	0	41.153	41.200	−0.047
20	−1	−1	1	−1	40.024	40.045	−0.021
21	−1	1	−1	1	40.087	39.964	0.123
22	0	0	0	0	41.890	41.789	0.101
23	−1	−1	−1	−1	39.813	39.830	−0.017
24	1	1	−1	1	39.902	39.877	0.025
25	1	−1	−1	−1	39.763	39.686	0.077
26	0	0	−1	0	41.455	41.535	−0.080
27	0	0	0	0	41.852	41.789	0.063
28	-1	1	1	1	39.062	39.134	-0.072

**Table 7:** Analysis of Variance was used to confirm the adequacy of the model used in this study.

Source	DF	Adj SS	Adj MS	F-Value	*p*-Value
**Model**	14	24.5007	1.75005	167.32	<0.001
**Residuals (error)**	13	0.1360	0.01046		
**Lack-of-Fit**	10	0.1303	0.01303	6.87	0.070
**Pure Error**	3	0.0057	0.00190		
**Total**	27	24.6366			

Note: *R^2^* = 99.45%; *Adj-R^2^* = 98.85%; *Pred-R^2^* = 97.04%. Abbreviations: DF = Degree of Freedom; SS = Sum of Square; MS = Mean Square.

**Table 8:** Regression coefficients significance: Coef, SE Coef, *t*-value, and *p*-value of the model terms (X_1_, X_2_, X_3_, X_4_) and their interactions are noted. The *t*- and *p*-values were determined by using *JMP 13* (SAS Institute, Wittington House, UK).

Model Term	Coef	SE Coef	*t*-Value	*p*-Value
Constant	41.7891	0.0354	1181.70	<0.001
X_1_	−0.0566	0.0241	−2.35	0.035
X_3_	−0.2557	0.0241	−10.61	<0.001
X_4_	−0.1524	0.0241	−6.32	<0.001
X_6_	0.0754	0.0241	3.13	0.008
X_1_*X_1_	−0.5328	0.0637	−8.37	<0.001
X_3_*X_3_	−0.9758	0.0637	−15.32	<0.001
X_4_*X_4_	−0.4068	0.0637	−6.39	<0.001
X_6_*X_6_	−0.1308	0.0637	−2.05	0.061
X_1_*X_3_	0.0133	0.0256	0.52	0.613
X_1_*X_4_	0.0014	0.0256	0.05	0.958
X_1_*X_6_	0.0008	0.0256	0.03	0.977
X_3_*X_4_	−0.2851	0.0256	−11.15	<0.001
X_3_*X_6_	−0.0557	0.0256	−2.18	0.048
X_4_*X_6_	0.0239	0.0256	0.93	0.367

Abbreviations: Coef = coefficient; SE Coef = standard error of the coefficient.

**Table 9:** Comparison of *S. griseus* recombinant β-glucosidase production under optimised and pre-optimised conditions. The non-optimised expression was carried out using basal medium (LB broth) overnight at 37 °C, 220 rpm and cells were induced with 1 mM IPTG when OD_600 nm_ reached 0.6.

Production Method	Fraction	Fraction Volume (mL)	Fraction Volume (mL)	Total Protein (mg)	Enzyme Activity U/mL	Total Activity (IU)
Optimised	Crude extract	10	9.94	99.40	42.00	420
Non-optimised	Crude extract	10	3.80	38.00	16.06	160

**Table 10:** *S. griseus* recombinant β-glucosidase purification table.

Purification Step	Total Protein (mg)	Total Activity (IU)	Specific Activity (IU/mg)	Yield (%)	Purification (Fold)
Crude extract	99.40	420	4.23	100	1.00
Ultrafiltration	71.82	392	5.46	93	1.29
Affinity chromatography	31.70	259	8.17	62	1.93
GST-tag cleavage	21.47	196	9.13	47	2.16

**Table 11:** The effect of metal ions or additive on purified *S.griseus* recombinant β-glucosidase was determined spectrometrically after 1-h and 6-h of incubation in the presence of 1 mM of each ion in potassium phosphate buffer, pH 7. The residual activity (%) was calculated in comparison to the activity obtained from enzyme in the same condition, but in the absence of any metal ion or additive. The results are the average of three independent experiments with standard derivation (±SD) noted (* *p*-value ≤ 0.05 and ** *p*-value ≤ 0.01 represent significant and very significant difference, respectively, based on two-tailed *t*-test).

Compounds	Residual Activity % ± SD (1 h)	Residual Activity % ± SD (6 h)
Control	100 ± 0.044	100 ± 0.074
Ca^2+^	105.57 ± 2.91	116.28 ± 3.90 **
Mg^2+^	106.55 ± 2.57	118.45 ± 4.11**
N^+^	101.98 ± 2.91	116.57 ± 1.54 **
K^+^	101.01 ± 1.65	113.53 ± 3.38 *
ZnSO_4_	86.99 ± 2.01 *	11.56 ± 0.58 **
(NH_4_)2S_4_	86.27 ± 1.36 *	99.74 ± 2.69

**Table 12:** Kinetic constants determined for purified β-glucosidase activity towards pNPG and cellobiose, as determined by non-linear regression analysis, using GraphPad Prism 7 (GraphPad Software, San Diego, CA, USA).

Substrate	K_m_ (mM)	V_max_ (μmol·min^−1^·mg^−1^)
*p*NPG	8.7 ± 0.42	243 ± 6.22
Cellobiose	15.8 ± 0.62	275 ± 7.12

**Table 13:** Kinetic parameters for β-glucosidases from various sources towards *p*NPG and cellobiose; n/d is not defined.

Microorganism	Substrate	Vmax	Km	Optimum Temp. and pH	References
*Thermoanaerobacterium thermosaccharolyticum*	pNPG	64 U/mg	0.62 mM	70 °C, pH 6.4	[56]
	cellobiose	120 U/mg	7.9 mM		
*Phoma sp.* KCTC11825BP	pNPG	n/d	0.3 mM	60 °C, pH 4.5	[61]
	cellobiose	n/d	3.2 mM,		
*Pyrococcus furiosus*	pNPG	700 U/mg	0.15 mM	105 °C, pH 5	[64]
	Celobiose	470 U/mg	20 mM		
*Thermoascus aurantiacus*	pNPG	n/d	0.1137 mM	80 °C, pH 4.5	[65]
	cellobiose	n/d	0.6370 mM		
*Aureobasidium pullulans* (NRRL Y-1297)	pNPG	897 μmol·min^−1^·mg^−1^	1.17 mM	75 °C, pH 4.5	[66]
	cellobiose	800 μmol·min^−1^·mg^−1^	1.00 mM		
*Monascus purpureus* NRRL1992	pNPG	6.51 U/mg	0.39 mM	50 °C, pH 5.5	[47]
	cellobiose	4.71 U/mg	2.86 mM		
*Aspergillus fumigatus* Z5	pNPG	141.60 μmol·min^−1^·mg^−1^	1.73 mM	60 °C, pH 6	[67]
	cellobiose	52.37 μmol·min^−1^·mg^−1^	1.75 mM		
*Stachybotrys* strain	pNPG	78 U/mg	0.27 mM	50 °C, pH 5	[68]
	cellobiose	59.4 U/mg	2.22 mM		
*Neosartorya fischeri* NRRL181	pNPG	886 μmol·min^−1^·mg^−1^	68 mM	40 °C, pH 6	[69]
*Aspergillus niger*	pNPG	166 μmol·min^−1^·mg^−1^	8.0 mM	50 °C, pH 8	[70]
